# Analysis of a Novel Entry Point for Freehand Ventriculostomy Using Computerized Tomography Scans

**DOI:** 10.7759/cureus.21079

**Published:** 2022-01-10

**Authors:** Anton N Konovalov, Vadim Gadzhiagaev, Alexei A Veselkov, Dmitry Okishev, Shalva Eliava

**Affiliations:** 1 Cerebrovascular Surgery, Burdenko Neurosurgical Center, Moscow, RUS; 2 Neurosurgery, Burdenko Neurosurgical Center, Moscow, RUS; 3 Neuro-Oncology, Burdenko Neurosurgical Center, Moscow, RUS; 4 Vascular Surgery, Burdenko Neurosurgical Center, Moscow, RUS

**Keywords:** freehand, anatomical, ct, hydrocephalus, evd

## Abstract

Background

External ventricular drain (EVD) placement is one of the most common procedures in neurosurgery. Neurosurgeons generally prefer to access the ventricles via Kocher’s point since it is the most common point of entry to this area; however, this point is used to describe different anatomic landmarks and is not well-defined.

Objective

The present study aims to describe and provide an anatomical assessment of a novel ventriculostomy access point developed by the authors using computerized tomography (CT) scans performed on 100 patients.

Materials and methods

Data were collected from 100 randomly selected patients with normal ventricular anatomy found on their 1.0 mm-slice CT scans performed at the Burdenko Neurosurgical Center from March 2019 to June 2021. The CT inclusion criteria were: CT slices < or = to 1 mm and absence of brain herniation. Patients with brain mass lesions, severe brain edema, and pneumocephalus were excluded. Age, gender, and ventricular size were not exclusion criteria.

Results

The mean patient age was 43.58 years (range 4-73), with 50 men and 50 women. The mean Evan’s index was 25.7 % (SD=4.38 %, range 10.2-41.0 %). No differences were found between the angles of EVD placement on either side (89.50±1.22 degrees on the right and 89.60±1.14 degrees on the left). Hence, nearly all EVD cases had been placed perpendicularly to the skull surface at a pinpoint location.

Conclusion

The proposed point of successful ventriculostomy placement in this study was 3 cm from the bregma along the coronal suture. The angle of EVD placement was approximately 90 degrees in almost all patients and was independent of the patient’s age and the side of the head that was entered. Little correlation was found between the value of the entry angle and Evan’s index. The point is simply identifiable, and its entry is easily accessible in practice.

## Introduction

External ventricular drain (EVD) placement is a common procedure in neurosurgical practice [1-2]. Neurosurgeons generally prefer to access the ventricles using Kocher’s point since it is the most common point of entry to this area; however, this point is used to describe different anatomic landmarks and is not well-defined [3-5]. Recently, new devices were invented to better navigate ventriculostomy placement and include manual devices that determine the angular placement with respect to the skull surface, endoscopy, a stereotactic neuronavigation system, ultrasonic navigation, MRI-assisted navigation, and smartphone software [6-10].

Due to logistic and economic reasons, especially in emergency cases, neuronavigation devices are not always available, thus, surgeons rely on craniometric point EVD placement [11]. This anatomy-based technique remains the most common and available method. Apart from Kocher’s point, others have been proposed such as Paine’s and Dandy’s points, etc. Each point has its advantages and disadvantages [4].

The present study aims to describe and provide an anatomical assessment of a novel ventriculostomy point developed by the authors of the present paper using computerized tomography (CT) scans from 100 patients.

## Materials and methods

All imaging and clinical data were extracted from the Burdenko Neurosurgical Center database. Thin-slice CT scans conducted at the Burdenko Neurosurgical Center from March 2019 to June 2021 were evaluated. Data were collected from 100 randomly selected patients with normal ventricular anatomy. The inclusion criteria were: CT slices < or = 1 mm thick, with the absence of brain herniation. Patients with brain mass lesions, severe brain edema, and pneumocephalus were excluded. Age, gender, and ventricular size were not exclusion criteria.

**Figure 1:**
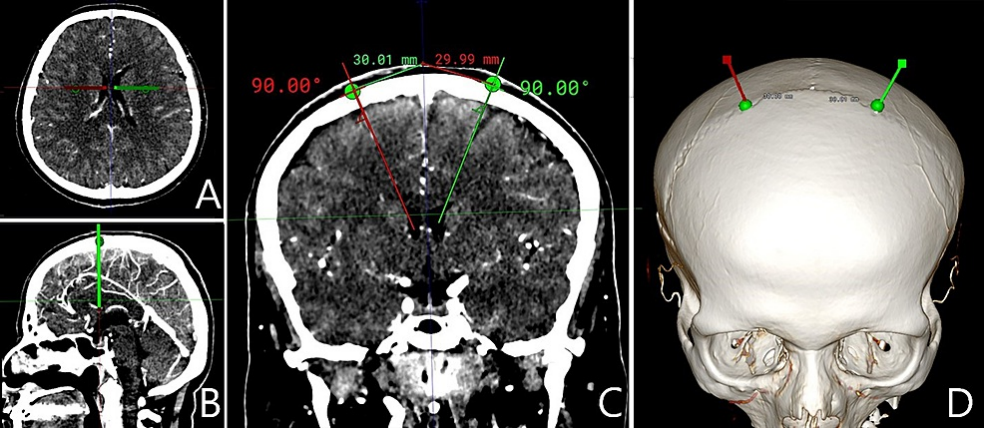
Entry point and EVD placement angle measurements on CT scan A, axial slices; B, trajectory of EVD insertion in the sagittal plane; C, trajectory of EVD insertion in the frontal plane; D, 3D reconstruction imaging depicting the point of trephination on the skull surface EVD = external ventricular drain; CT = computed tomography

**Figure 2:**
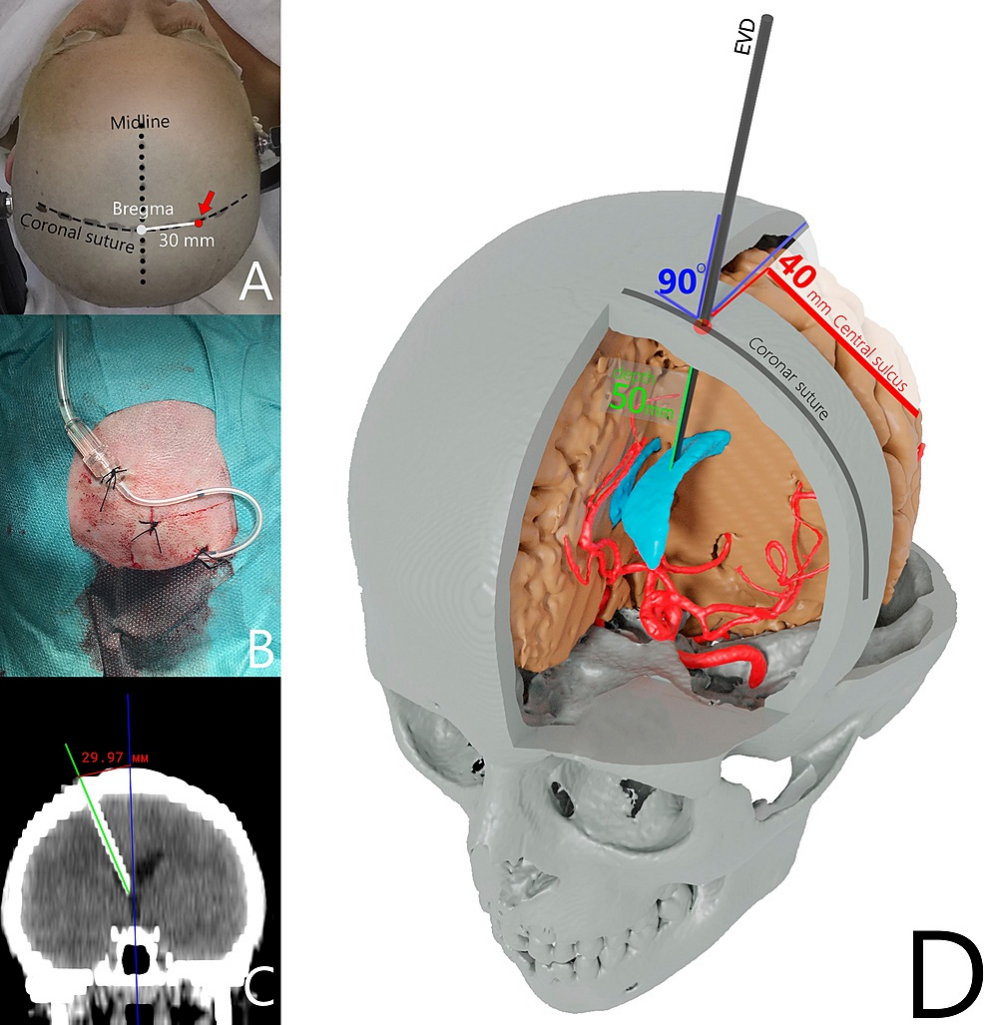
Case example of EVD placement A, B, C - Case example of an EVD placement at a point along the coronal suture 3 cm from the bregma; D – schematic illustration of EVD placement at the proposed point EVD = external ventricular drain

Inobitec Dicom Viewer Software (Inobitec LLC, Voronezh, Russia) was used in the multiplanar reconstruction (MPR) mode and 3D reconstruction (Figure 1). A specific entry point was marked on the coronal suture within 3 cm of 3D images of the bregma, and this was done on both sides. A frontal plane crossing these points was set up in MPR mode. Next, one line was placed from the body of the lateral ventricle to the indicated entry point marker. The second line was tangential to the circle of the outer bone plate and crossed the pinpointed entry site. The Inobitec Cobb Angle function tool (Inobitec LLC) was applied to measure the angle between these two lines. This angle was the angle of EVD placement. Evan’s index was calculated in all patients, consistent with the standard formula based on axial CT slices. The methodology for measurements was the same in all cases. Figure 2 shows a case example of EVD placement at this point.

Statistical analysis was performed using jamovi software, version 1.6 (https://www.jamovi.org/download.html). Shapiro-Wilk’s test and Q-Q plot analysis were used to assess the variables for normality. The non-parametric one-way analysis of variance (ANOVA) test (Kruskal-Wallis test) was used to detect any significant difference in Evan’s index and the angle between age groups by means of Dwass-Steel-Critchlow-Fligner pairwise comparisons. The non-parametric Friedman’s test was implemented to compare entry angles on the left and right sides. The relationship between Evan’s index and the entry angle was assessed by the analysis of covariance (ANCOVA).

## Results

CT scans of 100 patients were evaluated (50 men and 50 women). The mean patient age was 43.58 years (range 4-73). All patients were distributed into five groups (0-10, 11-20, 21-40, 41-60, and >60 years of age) for further analysis. No differences were found between the angles on either side (89.50±1.22 degrees on the right and 89.60±1.14 degrees on the left; χ2=0.0909; p=0.763). Mean placement angles from both sides were calculated for each patient and used for further analysis since no differences had been found between the angles of EVD placements on the right or left. The mean angle of EVD placement comprised 89.5 degrees. Hence, nearly all EVD catheters were supposed to be placed perpendicularly to the skull surface at a pinpointed access site.

**Figure 3:**
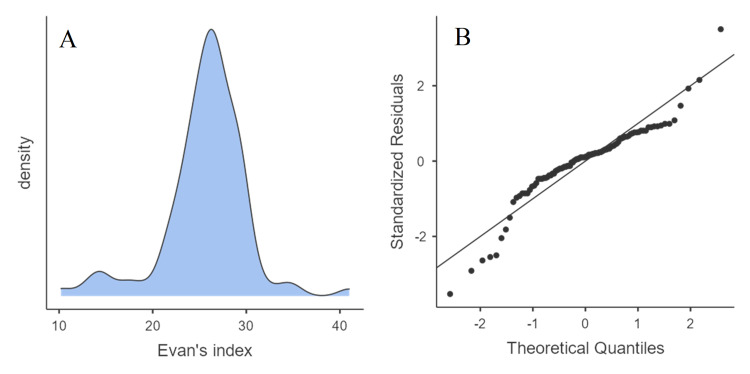
Density plot (A) and Q-Q plot (B) depicting the distribution of Evan’s index in the overall group

The mean Evan’s index was 25.7 % (SD=4.38 %; range 10.2-41.0 %). Although Evan’s index distribution showed some signs of normality on the density plot and Q-Q plot (Figure 3), it failed Shapiro-Wilk’s test (W=0.906; p<0.001), and thus non-parametric tests were used for further evaluation.

The Kruskal-Wallis test showed a significant difference in Evan’s index between age groups (χ2=10.02; p=0.040). However, in pairwise comparisons, none of the findings reached statistical significance. No significant difference was found in the ventriculostomy placement angle between age groups, both in the Kruskal-Wallis test (χ2=3.37; p=0.498) and in pairwise comparisons.

**Figure 4:**
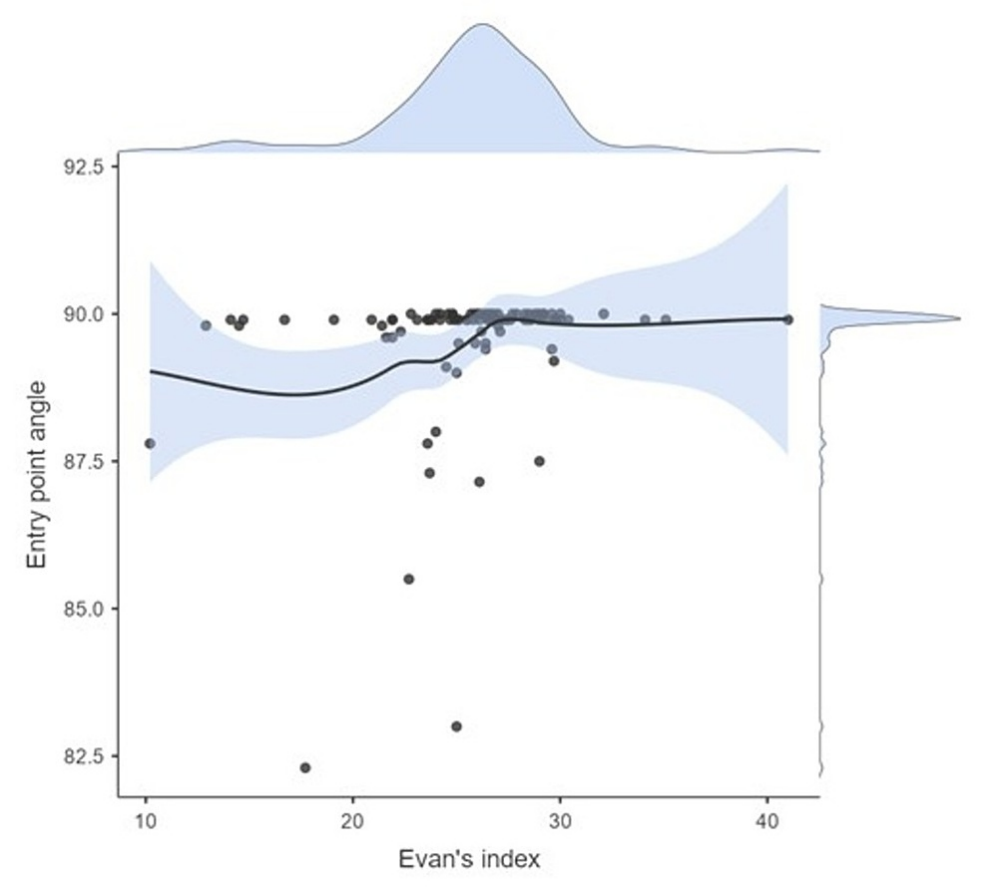
Regression line between the entry angle and Evan's index and their respective density plots

The EVD entry angle was < 89 degrees in 9% of the cases. However, it was 82.3 degrees in 1% of the cases. We performed an ANCOVA test of the entry angle value of different age groups using Evan’s index as a covariate (Table 1). We found that Evan’s index correlated significantly with the entry angle (F=6.97; p=0.010); however, only to a minor extent (η2p=0.073). Figure 4 shows the regression line between the entry angle and Evans’s index as well as their density plots. No effect on entry angles was found for various age groups and the interaction of age group and Evan's index (Table 1).

**Table 1:** ANCOVA test of the entry angle of different age groups using Evan’s index as a covariate ANCOVA = analysis of covariance

		Sum of squares		df		Mean square		F		p		η²p	
Overall model		28.06		9		3.12		1.50		0.160			
Evan's index		10.05		1		10.05		6.97		0.010		0.073	
Age group		9.10		4		2.27		1.58		0.187		0.066	
Age group ✻ Evan's index		8.91		4		2.23		1.55		0.196		0.065	

## Discussion

The EVD placement methodology differs among specialists. Numerous studies (Table 2) focusing on this topic are published annually. A standardized methodology is still not well-defined, however, and further investigation is required. It is worth noting that EVDs are placed more than 500 times annually in high-volume centers such as ours. In such circumstances, neuronavigation is not often used, and EVD placement relies on craniometric points.

**Table 2:** Common entry points proposed for an EVD placement EVD = external ventricular drain

Point name	Landmarks	Depth of insertion	Target point of insertion	Accuracy, %
Hildebrandt, Ghajar [12,16]	11 cm superior and posterior from the nasion and 3 cm lateral to the midline	6 cm below the skin surface	Frontal horn of the ipsilateral lateral ventricle	60-96%
Kaufman [17]	5 cm superior to the nasion and 3 cm lateral to the midline	7 cm below the surface of the skin	Within the frontal horn of the ipsilateral lateral ventricle	90%
Paine, Park [18,19]	2.5 cm above the floor of the anterior cranial fossa and 2.5 cm anterior to the Sylvian fissure	4 to 5 cm below the surface of the brain	Frontal horn of the ipsilateral ventricle	94%
Menovsky [20]	After completing the initial exposure and drilling the keyhole burr hole, the dura should be incised. The ventricular catheter should be passed through the burr hole and directed 45 degrees toward the midline and 20 degrees superior to the orbitomeatal line.	5 to 6.5 cm below the surface of the dura	Frontal horn of the ipsilateral lateral ventricle	87%
Tubbs [21]	The needle tip is placed under the upper left or right eyelid and advanced at a trajectory that is 45 degrees superior to the orbitomeatal line and 20 degrees to the midline.	8 cm below the surface of the skin	Frontal horn of the ipsilateral lateral ventricle	No clinical series determining its accuracy has been completed
Frazier [22]	6 cm superior to the inion and 3 to 4 cm left or right to the midline	5 cm below the surface of the brain	The body of the ipsilateral lateral ventricle	100%
Dandy [23]	3 cm above the inion and 2 cm left or right of the midline	4 to 5 cm below the surface of the dura	Body of the ipsilateral lateral ventricle	100%
Konovalov [24]	25-30 mm from the midline on the coronal suture	6 cm below the skin surface	The body of the ipsilateral lateral ventricle	93%

Kocher’s point, named after Emil Theodor Kocher, is a traditional point for EVD placement [12]. Kocher’s point is defined as a point 2-3 cm from the midline and 10-12 cm from the glabella or 1 cm anterior to the coronal suture. However, many different points for ventriculostomy were proposed (Table 2), which created anatomical uncertainty and confused neurosurgeons who were performing EVD placements. Despite the fact that Kocher’s point is the most applicable, the prevalence of EVD misplacement is about 11.7%-40% according to some studies [1,13-14]. In these cases, a catheter is usually placed in the thalamus, basal ganglia, internal capsule, brainstem, and other eloquent areas. According to Rehman et al., 10.4% of imaginary catheters passing through Kocher’s point ended up in a non-ventricular space and 20.8% in the contralateral ventricle [15]. Thus, the trajectory should be adjusted when an EVD is placed through Kocher’s point.

Our proposed point is easily identified based on standard anatomic landmarks, and the EVD trajectory placement is simple to accomplish. The EVD entry point is located 3 cm from the bregma along the coronal suture. The coronal suture and bregma are palpated as a bulge in the frontal area behind the hairline. Another landmark for the bregma is its 12-13 cm location from the nasion. A trephination is performed strictly perpendicular to the outer bone plate. Following these steps, an EVD is easily placed independent of a neurosurgeon's prior experience, as confirmed by a previous study [24]. The dura mater is firmly fixed to the inner bone plate at the cranial sutures, such that EVD placement on the coronal suture precludes an epidural hematoma. Approximately 3 cm from the bregma is a safe entry zone and not associated with an injury involving the parasagittal veins and eloquent motor cortex, the latter of which is normally located 4-5 cm behind the coronal suture. EVD placement was not associated with an optimal target insertion site using points further lateral than 3 cm on the coronal suture in a set of perpendicular insertions [24].

In the study by Konovalov et al., EVD placement was highly effective in 13 patients (92.8%) without any hemorrhagic events, when the proposed technique was applied [24]. CT scan analysis conducted in the study showed that the required angle of EVD placement was the same, independent of individual skull anatomy and ventricular size. In patients with increased Evan’s index, a direct correlation is noted with the entry angle. In the case of slit ventricles, an EVD is also placed perpendicularly. When the required angle is < 90 degrees, according to the CT scan, the additional landmark of the midline should be implicated. The frontal plane traversing the coronal suture typically crosses the anterior part of the body of the lateral ventricle. The ventricular system is positioned strictly at the midline 5 cm deep to the inner bone plate. Therefore, independent of bone surface or ventricular size, an EVD can be placed by relying on the midline as a landmark.

Every technique has its pros and cons [4]. Our method is easily conducted, leading to efficient EVD placement. The associated risk, however, is not greater as described in other studies.

Our study faces the following limitations: it relies only on radiographic data; therefore, comparison with clinical studies is not directly possible. Nevertheless, this study demonstrates our clinical experience and techniques in the anatomical assessment of novel ventriculostomy entry points using CT.

## Conclusions

The proposed point of entry for EVD placement is located 3 cm from the bregma along the coronal suture. The angle of EVD placement does not depend on the patient’s age or side of the head. It comprised approximately 90 degrees in all cases. There is little correlation between the value of the angle and Evan’s index, which can be considered when analyzing a patient’s brain imaging before EVD placement. As such, if a trephination is placed perpendicular to the bone surface, an EVD catheter is placed into the body of the lateral ventricle, as shown in our clinical study. The point is simply identifiable and easily applicable in practice.
